# Supplementary figures and images for: Elucidating the diet of the island flying fox (Pteropus hypomelanus) in Peninsular Malaysia through Illumina Next-Generation Sequencing
Source: PeerJ. 2017 Apr 12;5:e3176. doi: 10.7717/peerj.3176 (PMC5391789; doi:10.7717/peerj.3176)

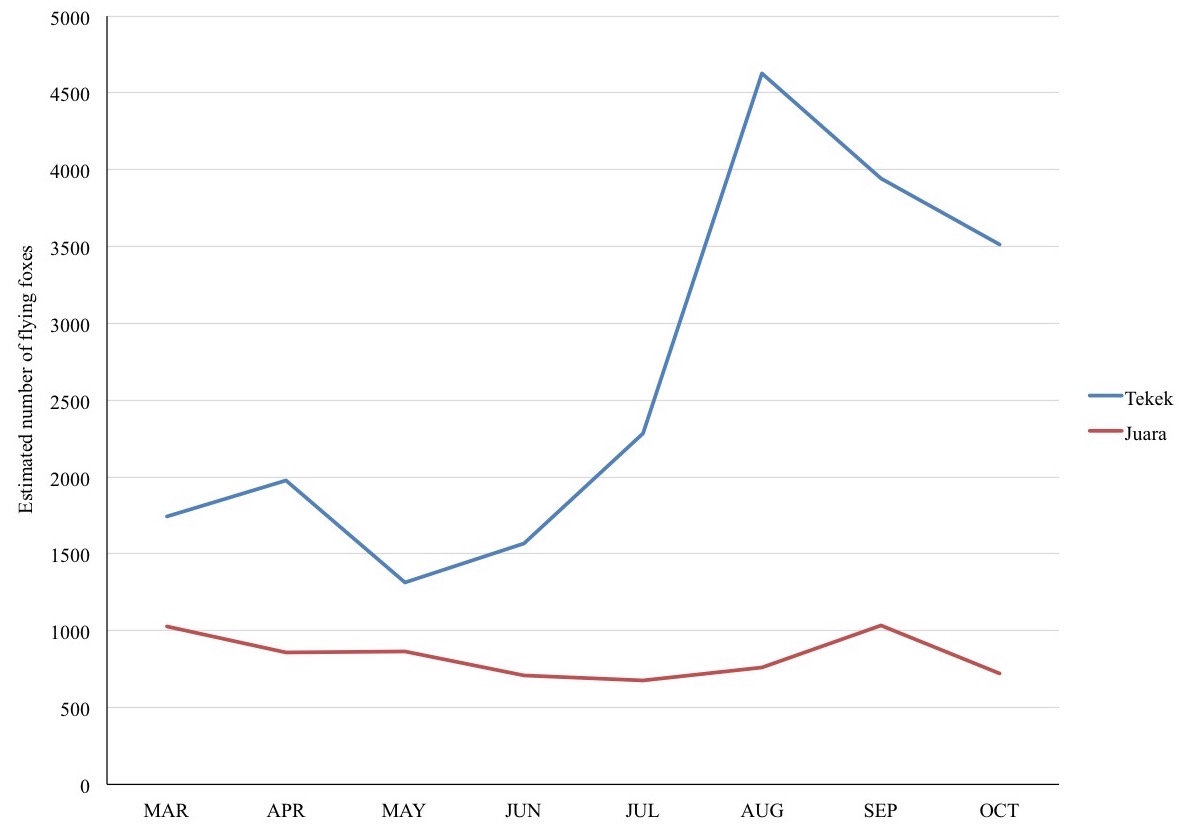

Supplement: Figure S1 — Monthly roost counts of flying foxes in Tekek and Juara villages between Mar and Oct 2015, Tioman Island. Due to the accessibility of the roosts, these counts were found to be more accurate than exit/emergence/fly-out counts. Exit counts were found to be impractical, as the bats did not fly out in steady streams towards specific directions, and no appropriate vantage points existed to be used as counting stations. [file peerj-05-3176-s001.jpg]

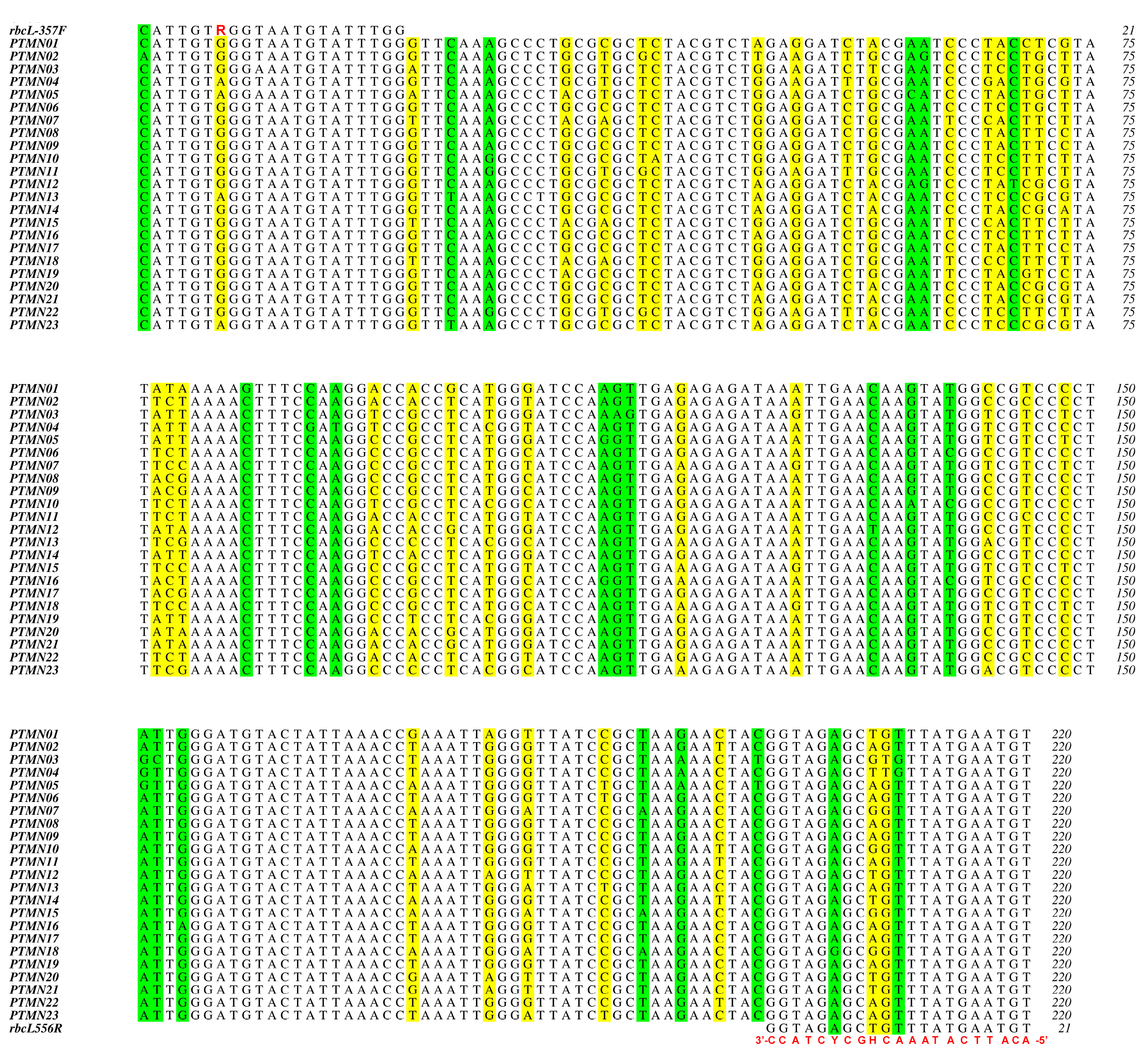

Supplement: Figure S2 — Residues that are divergent across all sequences are highlighted in green (>90% similarity) and yellow (<90% similarity). [file peerj-05-3176-s002.png]

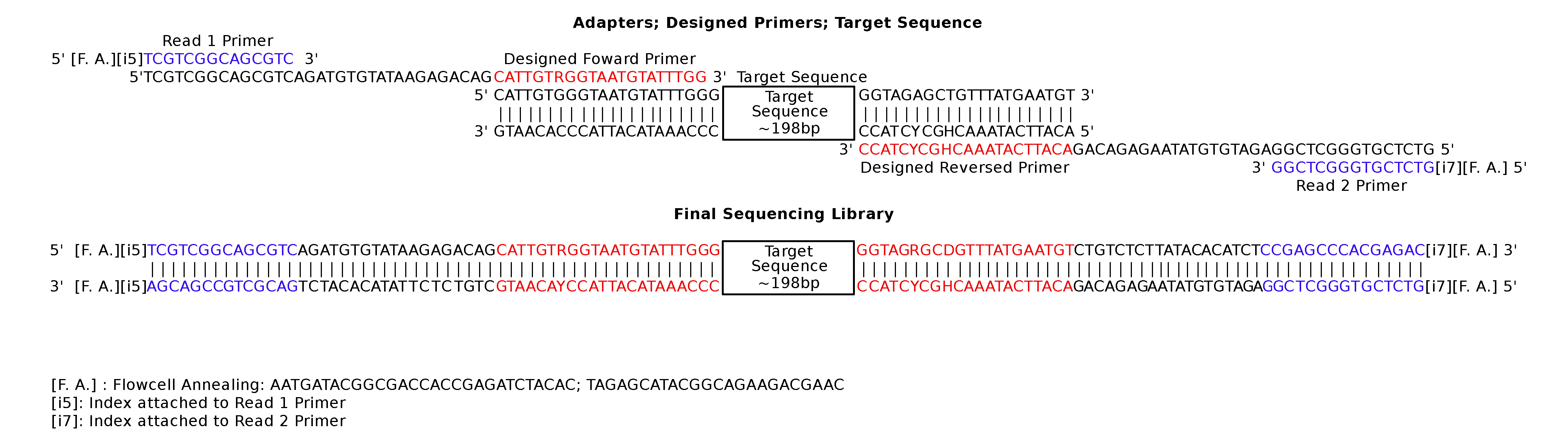

Supplement: Figure S3 [file peerj-05-3176-s003.png]

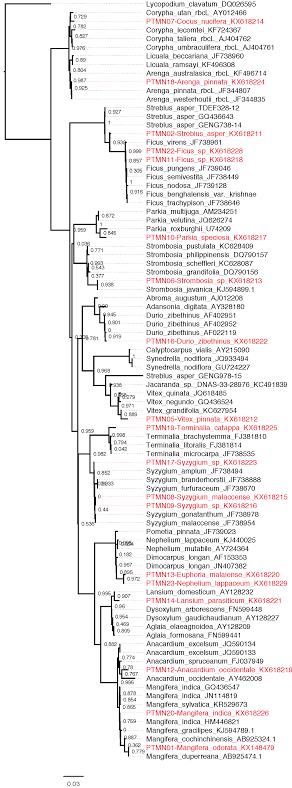

Supplement: Figure S4 — Maximum likelihood phylogenetic tree depicting the evolutionary relationship among rbcL sequences obtained from individually collected leaf samples (red-coloured tips) of potential flying fox food plants and rbcL sequences from online reference databases. PTMN codes refer to barcodes generated in this study and accession numbers are shown next to each taxon. Values in nodes indicate SH-like local support values. Scale bar indicates number of substitution per site. [file peerj-05-3176-s004.png]

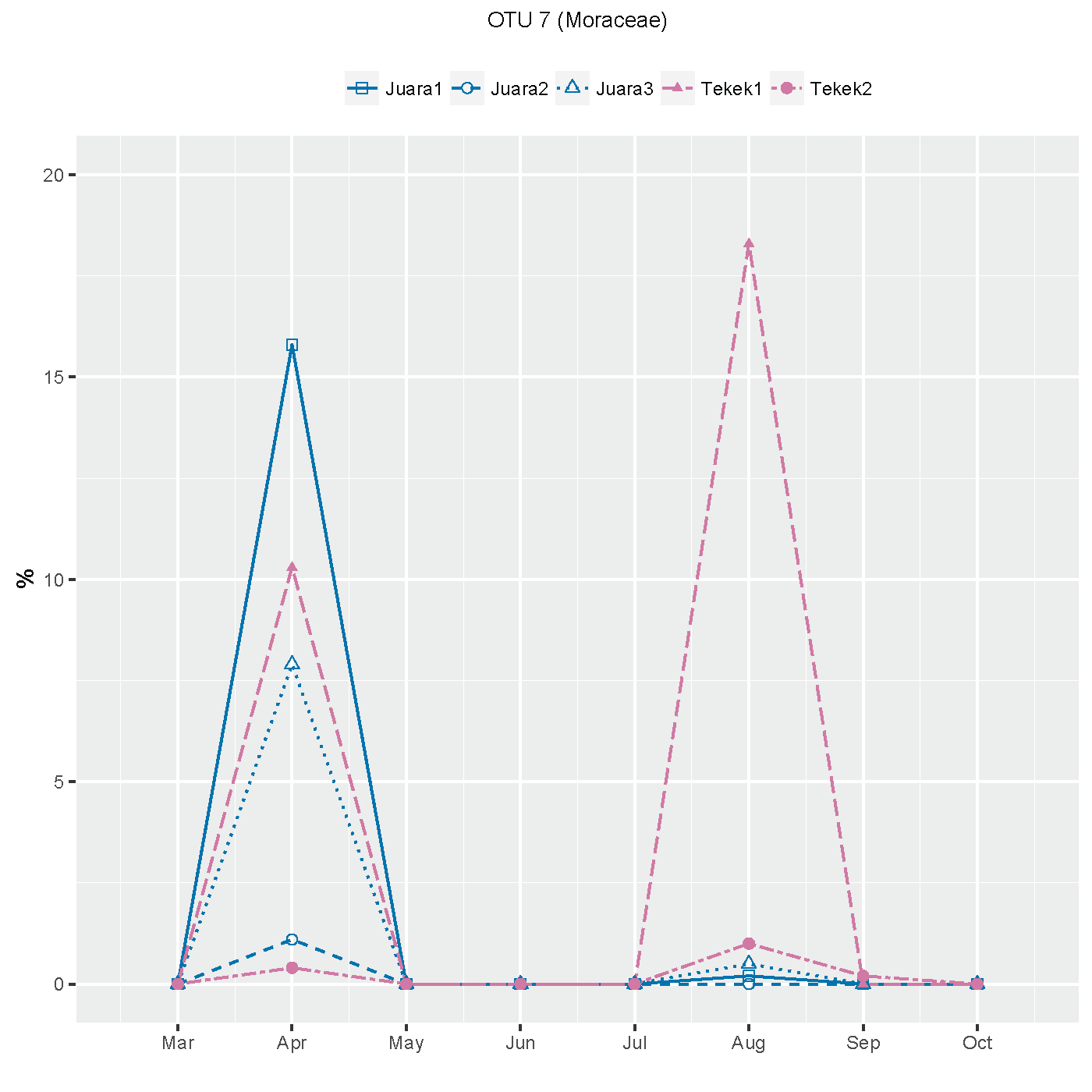

Supplement: Figure S5 [file peerj-05-3176-s005.png]
