# Supplementary material for: Elucidating the diet of the island flying fox (Pteropus hypomelanus) in Peninsular Malaysia through Illumina Next-Generation Sequencing
Source: PeerJ. 2017 Apr 12;5:e3176. doi: 10.7717/peerj.3176 (PMC5391789; doi:10.7717/peerj.3176)
Supplement: Table S1 — Summary information of 19 potential flying fox food plant reference specimens obtained from Tioman Island, Peninsular Malaysia: identification of plant specimen based on morphology, genus match(tick)/non-match(cross) based on rbcL sequences (Fig. 3), approximate GPS coordinates where the plant specimens were collected, their specimen code and GenBank accession codes. [file peerj-05-3176-s006.docx]

**Supplementary Table S1:** Summary information of 19 potential flying fox food plant reference specimens obtained from Tioman Island, Peninsular Malaysia: identification of plant specimen based on morphology, genus match(tick)/non-match(cross) based on *rbcL* sequences (Fig. 3), approximate GPS coordinates where the plant specimens were collected, their specimen code and GenBank accession codes.

| **Morphology-based ID of plant specimens** | **Genus match based on rbcL sequences** | **GPS coordinates** | **Specimen code** | **GenBank acccession code** |
| --- | --- | --- | --- | --- |
| *Anacardium occidentale* | ✓ | N2° 47.756' E104° 12.220' | PTMN12 | KX618219 |
| *Arenga pinnata* | ✓ | N2° 48.048' E104° 11.823' | PTMN18 | KX618224 |
| *Cocus nucifera* | ✗ | N2° 47.652' E104° 12.176' | PTMN07 | KX618214 |
| *Durio zibethinus* | ✓ | N2° 47.462' E104° 12.047' | PTMN16 | KX618222 |
| *Euphoria malaiense* | ✗ | N2° 47.300' E104° 12.139' | PTMN13 | KX618220 |
| *Ficus* sp. 1 | ✓ | N2° 48.197' E104° 11.566' | PTMN11 | KX618218 |
| *Ficus* sp. 2 | ✓ | N2° 49.354' E104° 10.145' | PTMN22 | KX618228 |
| *Lansium parasiticum* | ✓ | N2° 48.012' E104° 11.906' | PTMN14 | KX618221 |
| *Mangifera indica* | ✓ | N2° 47.645' E104° 12.176' | PTMN20 | KX618226 |
| *Mangifera odorata* | ✓ | N2° 48.134' E104° 11.745' | PTMN01 | KX148479 |
| *Nephelium lappaceum* | ✗ | N2° 49.353' E104° 09.916' | PTMN23 | KX618229 |
| *Parkia speciosa* | ✓ | N2° 48.595' E104° 10.758' | PTMN10 | KX618217 |
| *Streblus asper* | ✗ | N2° 26.214' E103° 50.857' | PTMN02 | KX618211 |
| *Strombosia* sp. | ✓ | N2° 48.737' E104° 10.537' | PTMN06 | KX618213 |
| *Syzygium malaccense* | ✓ | N2° 47.406' E104° 12.096' | PTMN08 | KX618215 |
| *Syzygium* sp. 1 | ✓ | N2° 47.740' E104° 12.218' | PTMN09 | KX618216 |
| *Syzygium* sp. 2 | ✓ | N2° 47.965' E104° 11.983' | PTMN17 | KX618223 |
| *Terminalia catappa* | ✓ | N2° 47.615' E104° 12.190' | PTMN19 | KX618225 |
| *Vitex pinnata* | ✓ | N2° 47.745' E104° 12.225' | PTMN05 | KX618212 |
