# Supplementary material for: Elucidating the diet of the island flying fox (Pteropus hypomelanus) in Peninsular Malaysia through Illumina Next-Generation Sequencing
Source: PeerJ. 2017 Apr 12;5:e3176. doi: 10.7717/peerj.3176 (PMC5391789; doi:10.7717/peerj.3176)
Supplement: Table S2 [file peerj-05-3176-s007.docx]

**Supplementary Table S2:** Primers used in this study for the amplification of *rbcL* gene fragment from flying fox droppings.

| **Primer** | **Sequence** | **Reference** |
| --- | --- | --- |
| rbcLaf-M13 | *TGTAAAACGACGGCCAGT***ATGTCACCACAAACAGAGACTAAAGC** | Kress and Erickson 2007 |
| rbcLa-revM13 | *CAGGAAACAGCTATGAC***GTAAAATCAAGTCCACCRCG** | Kress and Erickson 2007 |
| rbcL-357F | **CATTGTRGGTAATGTATTTGG** | This study |
| rbcL-556R | **ACATTCATAAACHGCYCTACC** | This study |
| IlluM-rbcLF | TCGTCGGCAGCGTCAGATGTGTATAAGAGACAG**CATTGTRGGTAATGTATTTGG** | This study |
| IlluM-rbcLR | GTCTCGTGGGCTCGGAGATGTGTATAAGAGACAG**ACATTCATAAACHGCYCTACC** | This study |

*Note: Bold letters - target sequence; underlined letters - Illumina partial adapter; italicized letters - M13 sequence.*
