# Supplementary material for: Elucidating the diet of the island flying fox (Pteropus hypomelanus) in Peninsular Malaysia through Illumina Next-Generation Sequencing
Source: PeerJ. 2017 Apr 12;5:e3176. doi: 10.7717/peerj.3176 (PMC5391789; doi:10.7717/peerj.3176)
Supplement: Table S3 [file peerj-05-3176-s008.docx]

**Supplementary Table S3:** Number of droppings per roost sampled from Juara (1406 droppings) and Tekek (1336 droppings) over 8 months on Tioman Island.

|  |  | **Juara** | | | | |  | **Tekek** | | |  |
| --- | --- | --- | --- | --- | --- | --- | --- | --- | --- | --- | --- |
| **Month** | **Date** | **R1** | **R2** | **R3** | **R4** | **R5** |  | **R1** | **R2** | **R3** | **Total** |
| Mar | 24 | 8 |  | 7 |  | 4 |  |  | 7 | 12 | *62* |
|  | 25 | 4 |  | 8 |  | 5 |  |  | 8 | 9 | *59* |
|  | 23 | 7 |  | 7 |  | 7 |  |  | 8 | 6 | *58* |
| *Subtotal* |  | *19* |  | *22* |  | *16* |  |  | *23* | *27* | *179* |
|  |  |  |  |  |  |  |  |  |  |  |  |
| Apr | 21 | 6 |  | 10 |  | 7 |  |  | 4 | 10 | *58* |
|  | 23 | 9 |  | 9 |  | 10 |  |  | 12 | 20 | *83* |
|  | 24 | 5 |  | 10 |  | 5 |  |  | 17 | 16 | *77* |
| *Subtotal* |  | *20* |  | *29* |  | *22* |  |  | *33* | *46* | *218* |
|  |  |  |  |  |  |  |  |  |  |  |  |
| May | 25 | 17 |  | 10 |  | 10 |  |  | 23 | 18 | *103* |
|  | 26 | 9 |  | 18 |  | 10 |  |  | 17 | 22 | *102* |
|  | 24 | 21 |  | 10 |  | 11 |  |  | 25 | 17 | *108* |
| *Subtotal* |  | *47* |  | *38* |  | *31* |  |  | *65* | *57* | *313* |
|  |  |  |  |  |  |  |  |  |  |  |  |
| Jun | 27 | 8 |  | 7 |  | 14 |  |  | 12 | 11 | *79* |
|  | 28 | 13 |  | 8 |  | 10 |  |  | 17 | 14 | *90* |
|  | 29 | 24 |  | 11 |  | 9 |  |  | 17 | 18 | *108* |
| *Subtotal* |  | *45* |  | *26* |  | *33* |  |  | *46* | *43* | *277* |
|  |  |  |  |  |  |  |  |  |  |  |  |
| Jul | 27 | 10 |  | 9 |  | 9 |  |  | 17 | 21 | *93* |
|  | 28 | 7 |  | 8 |  | 5 |  |  | 27 | 15 | *90* |
|  | 29 | 9 |  | 7 |  | 11 |  |  | 20 | 17 | *93* |
| *Subtotal* |  | *26* |  | *24* |  | *25* |  |  | *64* | *53* | *276* |
|  |  |  |  |  |  |  |  |  |  |  |  |
| Aug | 27 | 19 |  | 7 | 13 |  |  |  | 14 | 11 | *91* |
|  | 28 | 13 |  | 10 | 10 |  |  |  | 24 | 13 | *98* |
|  | 29 | 17 |  | 9 | 13 |  |  |  | 24 | 12 | *104* |
| *Subtotal* |  | *49* |  | *26* | *36* |  |  |  | *62* | *36* | *293* |
|  |  |  |  |  |  |  |  |  |  |  |  |
| Sep^ | 22 | 28 | 12 |  | 11 |  |  | 15 | 28 |  | 116 |
|  | 23 | 14 | 15 |  | 9 |  |  | 24 | 31 |  | *116* |
| *Subtotal* |  | *42* | *27* |  | *20* |  |  | *39* | *59* |  | *232* |
|  |  |  |  |  |  |  |  |  |  |  |  |
| Oct^ | 27 | 9 |  | 17 | 12 |  |  |  | 4 | * | *42* |
|  | 28 | 9 |  | 22 | 11 |  |  |  | 11 | * | *53* |
| *Subtotal* |  | *18* |  | *39* | *23* |  |  |  | *15* |  | *95* |

**Samples could not be collected due to rain.*

*^ Months with less than three days of sampling due to rain.*
